# Electricity and Dust

**Published:** 1897-07-10

**Authors:** 


### ELECTRICITY AND DUST.

Shoreditch has done well to institute a destructor by which to get rid of its "dust," the rubbish of all sorts which accumulates in every town in such enormous quantities as to become a perfect nightmare to those responsible for municipal administration.

The immense amount of material which has to be annually got rid of is almost as astonishing as the exceedingly primitive methods adopted by most of the London vestries for the purpose. Some simply send it out of their districts by barge or by rail—in other words, simply pass on the nuisance to someone else; others sort it, and sift it, and try to get out of it everything of any value, and then send it away; others merely dump it on vacant land for the delectation of the rats and other vermin of the neighbourhood; while a few, probably not more than half a dozen, burn it in destructors, or at any rate, burn as much of it as the apparatus at their disposal will allow them to deal with. Provincial towns have been far ahead of London in this matter, and it is from them that most of our experience as to the use of destructors has been gained.

The problem is by no means so simple as it looks. That all the offensive matters contained in "dust" can be destroyed by burning is plain enough, but to make the dust itself produce a fire sufficiently hot to consume all the vapours produced in its combustion is by no means an easy matter, as is shown by the many failures which have occurred.

The actual fuel value of London dust is but small, and no slight ingenuity is required to make it burn at all without giving off foul vapours. The first effect of making the stuff hot is to give rise to the vilest smells, and unless all the gases so produced are made to go through a really hot fire, they escape unburnt and cause a nuisance. This difficulty, however, can be at once overcome by the use of a certain amount of coal generally used in a secondary furnace, which, however, is a very wasteful proceeding, unless the heat so produced can be employed for some useful purpose. It is here that the advantage of the Shoreditch plan comes in. The papers have descanted with much delight upon the wonder of being able to put in garbage at one end of the apparatus and get out electricity at the other. But there is nothing wonderful about that. Given a source of heat, however produced, and there is no difficulty, by the help of a boiler, in producing power.

What, however, is really good, from a sanitary point of view, in the Shoreditch plan of associating dust destruction with the electric light producing industry, is that it enables coal to be used if required without wastefulness. The success of the scheme seems to depend on obtaining a large market for the electricity produced, for the smaller the proportion which the garbage can be made to bear to the total fuel used, the greater will be the facility of ensuring its complete combustion.

